# Supplementary material for: Identification of Entomopathogenic Fungi as Naturally Occurring Enemies of the Invasive Oak Lace Bug, Corythucha arcuata (Say) (Hemiptera: Tingidae)
Source: Insects. 2020 Oct 7;11(10):679. doi: 10.3390/insects11100679 (PMC7600444; doi:10.3390/insects11100679)
Supplement: Supplementary file 1 [file insects-11-00679-s001.pdf]

Table S1. Genbank accession numbers of sequences used in the phylogenetic analysis.

**Species**	**Voucher Information**	**ITS**	**SSU**	**LSU**	**TEF**	**RPB1**	**RPB2**	**Bloc**
Beauveria pseudobassiana	BBNK1	MT004821						MT001936
Lecanicillium pissodis	BBNK2	MT004822	MT004817	MT004833	MT027501	MT027505		
Beauveria pseudobassiana	BBC1	MT004823						MT001937
Beauveria pseudobassiana	BBC2	MT004824						MT001938
Beauveria pseudobassiana	BBC3	MT004825						MT001939
Beauveria pseudobassiana	BBC4	MT004826						MT001940
Samsoniella alboaurantia	BBC5	MT004827	MT004818	MT004834	MT027502		MT027508	
Beauveria pseudobassiana	BBC6	MT004828						MT001941
Lecanicillium pissodis	BBC7	MT004829	MT004819	MT004835	MT027503	MT027506	MT027509	
Beauveria pseudobassiana	BBC8	MT004830						MT001942
Akanthomyces attenuatus	BBC9	MT004831	MT004820		MT027504	MT027507	MT027510	
Beauveria pseudobassiana	BBC10	MT004832						MT001943
Beauveria medogensis	CGMCC 3.15617	KU994837			KU994833	KU994835	KU994834	KU994836
Beauveria bassiana	ARSEF 1040	AY531972			AY531881	HQ880830	HQ880902	HQ880689
Beauveria bassiana	ARSEF 1564	HQ880761			HQ880974	HQ880833	HQ880905	HQ880692
Beauveria bassiana	ARSEF 1848	AY531995			AY531904	HQ880832	HQ880904	HQ880696
Beauveria australis	ARSEF 4622	HQ880790			HQ880996	HQ880862	HQ880934	HQ880721
Beauveria australis	ARSEF 4580	HQ880788			HQ880994	HQ880860	HQ880932	HQ880719
Beauveria australis	ARSEF 4598	HQ880789			HQ880995	HQ880861	HQ880933	HQ880720
Beauveria brongniartii	ARSEF 617	HQ880782			HQ880991	HQ880854	HQ880926	HQ880713
Beauveria brongniartii	ARSEF 985	HQ880768			HQ880978	HQ880840	HQ880912	HQ880699

Beauveria brongniartii	ARSEF 10277	HQ880780			HQ880989	HQ880852	HQ880924	HQ880711
Beauveria asiatica	ARSEF 4384	AY532026			AY531935	HQ880857	HQ880929	HQ880716
Beauveria asiatica	ARSEF 4474	HQ880786			AY531936	HQ880858	HQ880930	HQ880717
Beauveria asiatica	ARSEF 4850	HQ880787			AY531937	HQ880859	HQ880931	HQ880718
Beauveria sungii	ARSEF 1685	AY531990			AY531899	HQ880881	HQ880953	HQ880740
Beauveria sungii	ARSEF 7279	HQ880813			HQ881009	HQ880885	HQ880957	HQ880744
Beauveria sungii	ARSEF 7280	HQ880814			HQ881010	HQ880886	HQ880958	HQ880745
Beauveria malawiensis	ARSEF 7760	DQ376247			DQ376246	HQ880897	HQ880969	HQ880756
Beauveria malawiensis	BCC 17613	HQ880824			HQ881016	HQ880896	HQ880968	HQ880755
Beauveria malawiensis	ARSEF 4755	HQ880825			HQ881015	HQ880895	HQ880967	HQ880754
Beauveria caledonica	ARSEF 7117	HQ880820			HQ881013	HQ880892	HQ880964	HQ880751
Beauveria caledonica	ARSEF 4302	HQ880821			HQ881014	HQ880893	HQ880965	HQ880752
Beauveria caledonica	AESEF 2567	AY532006			AY531915	HQ880889	HQ880961	HQ880748
Beauveria amorpha	ARSEF 2641	AY532008			AY531917	HQ880880	HQ880952	HQ880739
Beauveria amorpha	ARSEF 4149	HQ880804			HQ881006	HQ880876	HQ880948	HQ880735
Beauveria pseudobassiana	ARSEF 1855	HQ880796			HQ880999	HQ880868	HQ880940	HQ880727
Beauveria pseudobassiana	ARSEF 3405	AY532022			AY531931	HQ880864	HQ880936	HQ880723
Beauveria pseudobassiana	ARSEF 4933	AY532029			AY531938	HQ880870	HQ880942	HQ880729
Beauveria varroae	ARSEF 2694	HQ880802			HQ881004	HQ880874	HQ880946	HQ880733
Beauveria varroae	ARSEF 8257	HQ880800			HQ881002	HQ880872	HQ880944	HQ880731
Beauveria varroae	ARSEF 8259	HQ880801			HQ881003	HQ880873	HQ880945	HQ880732
Beauveria kipukae	ARSEF 7032	HQ880803			HQ881005	HQ880875	HQ880947	HQ880734
Beauveria vermiconia	AESEF 2922	AY532012			AY531920	HQ880894	HQ880966	HQ880753
Beauveria rudraprayagi	MTCC 8017	JQ266173			JQ990914	JQ990892	JQ990870	JQ990848
Beauveria lii	RCEF 5500	JN689372			JN689371	JN689374	JN689370	JN689373
Beauveria hoplocheli	Bt96	KC339697			KC339709	KM453950	KM453959	KM453974
Beauveria hoplocheli	Bt121	KC339687			KC339704	KM453956	KM453965	KM453968

Beauveria hoplocheli	Bt128	KC339693			KC339705	KM453952	KM453961	KM453972
Cordyceps militaris	ARSEF 5050	HQ880829			HQ881020	HQ880901	HQ880973	
Akanthomyces aculeatus	HUA 186145		MF416572	MF416520	MF416465			
Akanthomyces aculeatus	TS 772	KC519371	KC519368	KC519370	KC519366			
Akanthomyces attenuatus	CBS402.78	AJ292434	AF339614	AF339565	EF468782	EF468888	EF468935	
Akanthomyces attenuatus	KACC42493		KM283756	KM283780	KM283804	KM283826	KM283846	
Akanthomyces coccidioperitheciatus	NHJ 6709	JN049865	EU369110	EU369042	EU369025	EU369067	EU369086	
Akanthomyces kanyawimiae	TBRC 7242	MF140751		MF140718	MF140838	MF140784	MF140808	
Akanthomyces kanyawimiae	TBRC 7244	MF140752		MF140716	MF140836			
Akanthomyces lecanii	CBS101247	JN049836	KM283770	KM283794	DQ522359	KM283837	KM283859	
Akanthomyces lecanii	CBS102067		KM283771	KM283795	KM283818	KM283838	KM283860	
Akanthomyces muscarius	CBS143.62		KM283774	KM283798	KM283821	KM283841	KM283863	
Akanthomyces pistillariaeformis =tuberculatus	HUA 186131		MF416573	MF416521	MF416466			
Akanthomyces sulphureus	TBRC 7248	MF140758		MF140722	MF140843	MF140787	MF140812	
Akanthomyces sulphureus	TBRC 7249	MF140757		MF140721	MF140842	MF140786	MF140734	
Akanthomyces thailandicus	TBRC 7245	MF140754			MF140839		MF140809	
Akanthomyces thailandicus	TBRC 7246	MF140755		MF140719	MF140840		MF140810	
Akanthomyces tuberculatus	BCC16819		MF416600	MF416546	MF416490	MF416647		
Akanthomyces waltergamsii	TBRC 7250	MF140749		MF140715	MF140835			
Akanthomyces waltergamsii	TBRC 7251	MF140747		MF140713	MF140833	MF140781	MF140805	
Ascopolyporus polychrous	P.C. 546			DQ118737	DQ118745	DQ127236		
Ascopolyporus villosus	ARSEF 6355			AY886544	DQ118750	DQ127241		
Beauveria brongniartii	BCC 16585	JN049867	JF415951	JF415967	JF416009	JN049885	JF415991	
Blackwellomyces cardinalis	OSC 93609		AY184973	AY184962	DQ522325	DQ522370	DQ522422	
Blackwellomyces pseudomilitaris	NBRC 101409	JN943305	JN941748	JN941393		JN992482		

Blackwellomyces pseudomilitaris	NBRC 101410	JN943307	JN941747	JN941394		JN992481		
Blackwellomyces cardinalis	OSC 93610	JN049843	AY184974	AY184963	EF469059	EF469088	EF469106	
Gibellula longispora	NHJ 12014		EU369098		EU369017	EU369055	EU369075	
Gibellula ratticaudata	ARSEF 1915	JN049837	DQ522562	DQ518777	DQ522360	DQ522408	DQ522467	
Gibellula sp.	NHJ 10788		EU369101	EU369036	EU369019	EU369058	EU369078	
Gibellula sp.	NHJ 5401		EU369102			EU369059	EU369079	
Gibellula sp.	NHJ 7859		EU369107			EU369064	EU369085	
Hevansia arachnophila	NHJ 10469		EU369090	EU369031	EU369008	EU369047		
Hevansia cinerea	NHJ 3510		EU369091		EU369009	EU369048	EU369070	
Hevansia nelumboides	BCC 41864	JN201871	JN201863	JN201873	JN201867			
Hevansia novoguineensis	NHJ 11923		EU369095	EU369032	EU369013	EU369052	EU369072	
Lecanicillium acerosum	CBS418.81	EF641893	KM283762	KM283786	KM283810	KM283832	KM283852	
Lecanicillium antillanum	CBS350.85	AJ292392	AF339585	AF339536	DQ522350	DQ522396	DQ522450	
Lecanicillium aphanocladii	CBS797.84		KM283763	KM283787	KM283811	KM283833	KM283853	
Lecanicillium aranearum	CBS726.73a	AJ292464	AF339586	AF339537	EF468781	EF468887	EF468934	
Lecanicillium araneicola	BTCC-F35	AB378506						
Lecanicillium araneogenum	GZU1031Lea		KX845705		KX845697	KX845699	KX845701	
Lecanicillium dimorphum	CBS345.37		KM283764	KM283788	KM283812	KM283834	KM283854	
Lecanicillium flavidum	CBS300.70D	EF641877	KM283765	KM283789	KM283813		KM283855	
Lecanicillium fusisporum	CBS164.70	AJ292428	KM283769	KM283793	KM283817	KM283836	KM283858	
Lecanicillium kalimantanense	BTCC-F23	AB360356						
Lecanicillium longisporum	CBS102072		KM283772	KM283796	KM283819	KM283839	KM283861	
Lecanicillium longisporum	CBS126.27		KM283773	KM283797	KM283820	KM283840	KM283862	
Lecanicillium nodulosum	IMI 338014R	EF513012						
Lecanicillium pissodis	CBS118231		KM283775	KM283799	KM283822	KM283842	KM283864	
Lecanicillium primulinum	JCM 18525	AB712266		AB712263				
Lecanicillium primulinum	JCM 18526	AB712267		AB712264				

Lecanicillium psalliotae	CBS101270		EF469128	EF469081	EF469066	EF469095	EF469113	
Lecanicillium psalliotae	CBS363.86		AF339608	AF339559	EF468784	EF468890		
Lecanicillium psalliotae	CBS532.81	JN049846	AF339609	AF339560	EF469067	EF469096	EF469112	
Lecanicillium restrictum	CCF5252	LT548279			LT626943			
Lecanicillium sabanense	JCHA5	KC633232	KC633251	KC875225	KC633266		KC633249	
Lecanicillium saksenae	IMI 179841	AJ292432						
Lecanicillium subprimulinum	HKAS99548	MG585314	MG585316	MG585315	MG585317			
Lecanicillium subprimulinum	HKAS99549	MG585318	MG585320	MG585319	MG585321			
Lecanicillium tenuipes	CBS309.85	JN036556	KM283778	KM283802	DQ522341	KM283844	KM283866	
Lecanicillium testudineum	UBOCC-A112180	LT992874			LT992868			
Lecanicillium testudineum	UBOCC-A116026	LT992871			LT992867			
Lecanicillium uredinophilum	KACC44082		KM283758	KM283782	KM283806	KM283828	KM283848	
Lecanicillium uredinophilum	KACC47756		KM283759	KM283783	KM283807	KM283829	KM283849	
Lecanicillium wallacei	CBS101237	EF641891	AY184978	AY184967	EF469073	EF469102	EF469119	
Lecanicillium fungicola var. aleophilum	CBS357.80	NR_111064	KM283767	KM283791	KM283815	KM283835	KM283856	
Lecanicillium fungicola var. fungicola	CBS992.69	NR_119653	KM283768	KM283792	KM283816		KM283857	
Paecilomyces hepiali	CGMCC 3.17103	NR_160318			KJ676100			
Paecilomyces hepiali	SJL0909	HM135170	HM135172	HM135171				
Samsoniella alboaurantium	CBS 240.32	AY624178	JF415958	JF415979	JF416019	JN049895	JF415999	
Samsoniella alboaurantium	CBS 262.58	MH857775		MH869308	JQ425685	MF416654	MF416448	
Samsoniella aurantia	TBRC 7271	MF140764		MF140728	MF140846	MF140791	MF140818	
Samsoniella aurantia	TBRC 7272	MF140763		MF140727	MF140845		MF140817	
Samsoniella inthanonensis	TBRC 7915	MF140761		MF140725	MF140849	MF140790	MF140815	
Samsoniella inthanonensis	TBRC 7916	MF140760		MF140724	MF140848	MF140789	MF140814	
Simplicillium lamellicola	CBS 116.25	AJ292393	AF339601	AF339552	DQ522356	DQ522404	DQ522462	

Simplicillium lanosoniveum	CBS 101267	AJ292395	AF339603	AF339554	DQ522357	DQ522405	DQ522463	
Simplicillium lanosoniveum	CBS 704.86	AJ292396	AF339602	AF339553	DQ522358	DQ522406	DQ522464	
Simplicillium obclavatum	CBS 311.74		AF339567	AF339517	EF468798			
